# Supplementary material for: Three-dimensional ultrafast charge-density-wave dynamics in CuTe
Source: Nat Commun. 2024 Mar 16;15:2386. doi: 10.1038/s41467-024-46615-y (PMC10944522; doi:10.1038/s41467-024-46615-y)
Supplement: Supplementary file 1 — Supplementary Information [file 41467_2024_46615_MOESM1_ESM.pdf]

Supplementary Information

Three-dimensional ultrafast charge-density-wave dynamics in CuTe

Supplementary Note 1: Collective modes resolved by the oscillated $\Delta R/R$ spectra

As discussed in the second paragraph of the main text, there are two collective modes of amplitudon $\Delta(t)$ and phason $\phi(t)$ in correlated electron systems, as shown in Supplementary Fig. 1. Recently, a phenomenological time-dependent Ginzburg-Landau (TDGL) model (for the details, please see Note 5 in Supplementary Information) was employed to account for the temperature dependence of the amplitudon and non-equilibrium dynamics upon photoexcitation [1]. The TDGL model describes the distortions of CDW amplitudon via classical equations of motion for the complex coordinates of the electronic order parameter (EOP, $\tilde{\Delta}$) with linear coupling to the bare phonons ($\tilde{\xi}_n$) as shown in Supplementary Fig. 2. This provides a versatile framework to include effects such as time-dependent perturbations of the potentials and higher spatial dimensions [2, 3].

In terms of pump-probe spectroscopy, the transient reflectivity changes ($\Delta R/R$) of opaque materials can be expressed by [4]

$$\frac{\Delta R}{R} = \frac{4\Delta n}{n^2-1}$$

where n is the refractive index of the materials, and "1" stands for the free-space refractive index. Δn is the pump-induced change in the refractive index n, which can be expressed as

$$\Delta n = \frac{\partial n}{\partial N}\Delta N + \frac{\partial n}{\partial T}\Delta T_L$$

where ΔN and ΔT_L are the pump-induced excess carrier density and lattice temperature change, respectively. After pumping, the ΔN of carrier density changes dominate the Δn and $\Delta R/R$ in the sub-ps timescale. Through the electron-phonon coupling, Δn and $\Delta R/R$ will be further dominated by the ΔT_L of lattice temperature change when the ΔN has substantially decreased and raises the lattice temperature T_L associated with the phonons. Additionally,

the oscillation feature of phonons (lattice vibration) in the time domain can be resolved by pump-probe spectroscopy with suitable time resolution and phase-matching conditions. Since the coupling between $\tilde{\Delta}$ and $\tilde{\xi}_n$, as shown in Supplementary Fig. 2, the oscillation components in $\Delta R/R$ are explicitly associated with the collective modes in CDW materials.

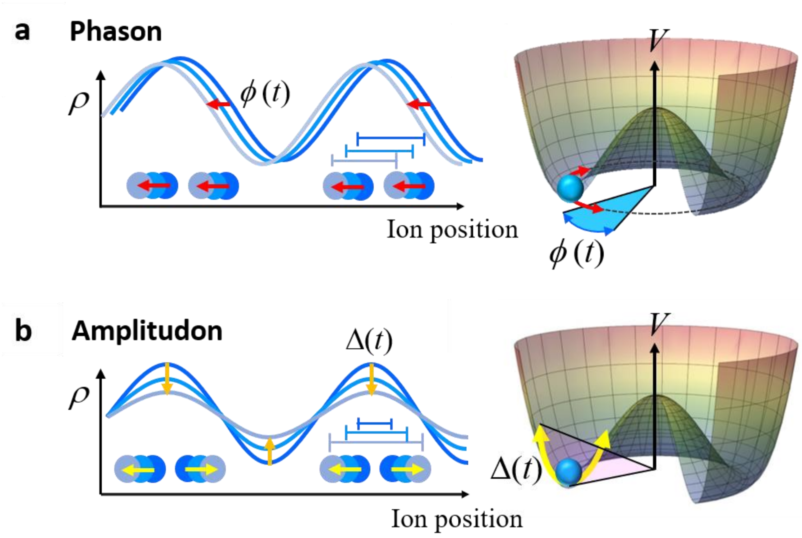

Supplementary Fig. 1. Schematics of spatial modulation (left) of ions/charge density (ρ) and phase space potentials (right) associated with the 1D crystal chain. **a** Phase mode (Phason) with the phase changes as a function of time, $\phi(t)$. **b** Amplitude mode (Amplitudon) with the potential changes as a function of time, $\Delta(t)$. The arrows indicate the tendency to change the ion's positions.

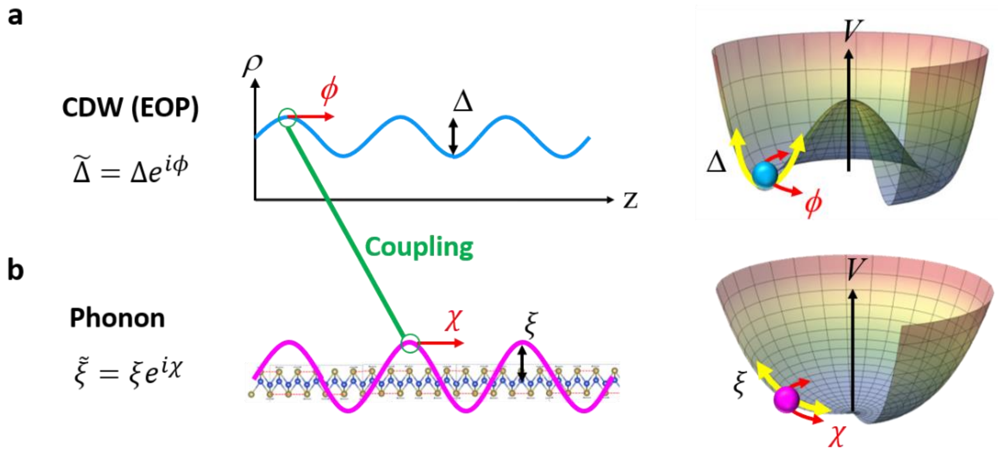

Supplementary Fig. 2. Schematics of spatial modulation (left) and phase space potentials (right) associated with **a** CDW (electronic order parameter, EOP) and **b** a single phonon with complex amplitudes $\tilde{\Delta}$ and $\tilde{\xi}$, respectively.

Supplementary Note 2: Ultrafast dynamics along the *b*-axis of CuTe

Supplementary Fig. 3a shows the typical $\Delta R/R$ spectra along the *b*-axis (i.e., $\theta = 8°$, ϕ_1 and $\phi_2 = 90°$ in Fig. 1) at various temperatures. Besides the large negative response, an oscillation component is observed at temperatures below 250 K. The negative response in $\Delta R/R$ spectra is fitted well using Eq. (1), e.g., at 35 K. By subtracting the exponential decay background (the dashed line in Supplementary Fig. 3a), we can extract the oscillation component from the $\Delta R/R$ spectra. After a fast Fourier transform (FFT), the FFT spectrum at 35 K is obtained, as shown in Supplementary Fig. 3b. Additionally, the oscillation component with a frequency of 1.64 THz is identified through the peak fitting.

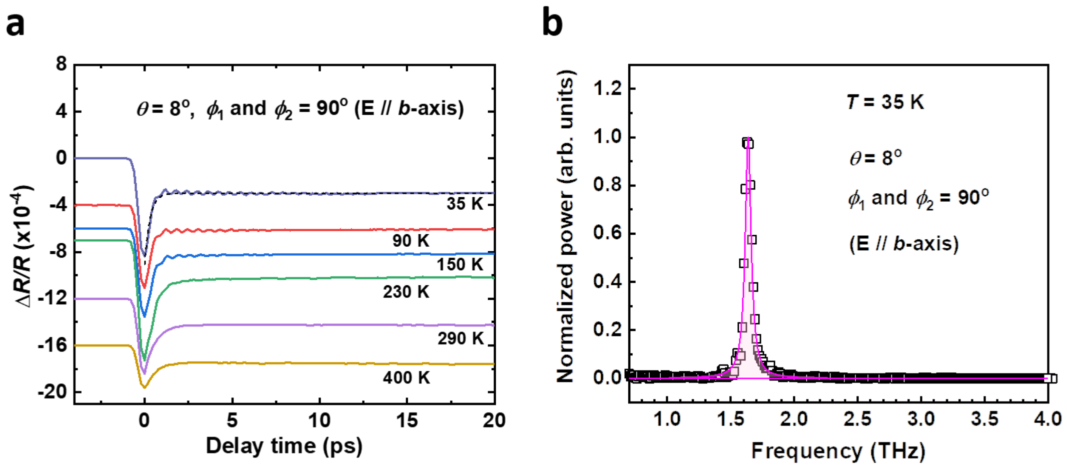

Supplementary Fig. 3. a $\Delta R/R$ along the *b*-axis (**E** // *b*-axis, i.e., $\theta = 8°$, ϕ_1 and $\phi_2 = 90°$) of a (001) CuTe single crystal at various temperatures. **b** A Fourier transform spectrum of the oscillation component at $T = 35$ K in **(a)**.

Supplementary Note 3: Raman spectra of CuTe

The temperature-dependent Raman measurements were performed on a home-built ultralow frequency Raman system using a 532 nm Nd:YAG DSSP laser (Lasos DPSSL GLK32150 TS) as an excitation light source. Using volume Bragg grating (VBG) filters allowed us to measure the Raman spectrum down to the filter cutoff frequency ±10 cm^{-1}. Meanwhile, the polarization-dependent Raman measurements can be performed by setting the excitation laser polarization and the detected Raman signal. A 50× objective (Olympus SLMPlan NA0.45) focused the excitation laser to a beam spot of ~1.5 μm with an on-sample power of ~1.9 mW. The Raman signal was collected by the same objective and sent through an optical fiber into the spectrometer (Horiba FHR640, 1200 grooves/mm grating, 100 μm entrance slit width, Symphony CCD). The sample was placed in a temperature-controlled cryostat (Linkam THMS350V) and purged with nitrogen.

Supplementary Fig. 4a shows the normalized temperature-dependent polarized Raman spectra of CuTe. In the PP configuration, the excitation and detection polarization were parallel to the *a*-axis, and the curves were normalized to the peak that falls between 0.60 ~ 1.80 THz (20 and 60 cm^{-1}). The Raman spectra show four modes when the temperature is decreased below 220 K. These modes are CDW-related modes and assigned with the name of amplitude (am) mode A_{am} (A_1, see Supplementary Fig. 7) with the frequency of 1.62 THz (54 cm^{-1}), and zone-folded (ZF) mode A_{ZF1} (A_{g}, see Supplementary Fig. 7) with the frequency of 2.25 THz (75 cm^{-1}), A_{ZF2} with the frequency of 3.03 THz (101 cm^{-1}), A_{ZF3} with the frequency of 3.57 THz (119 cm^{-1}) (e.g., the 80-K spectra in Supplementary Fig. 4a). The three zone-folded modes can be attributed to Raman-active phonons activated in the CDW state due to the folding of the Brillouin zone, consistent with the Raman spectra measured at 10 K in previous experimental reports [5]. Note that no Raman features were observed in the cross-polarization detection. The 1.62-THz (54 cm^{-1}) A_{am} peak feature at 80 K gets broadened and ref-shifted. The curve-fitted peak centers of these two peak features are shown as a function of temperature in Fig. 3a. Like A_{ZF1}, the A_{ZF2} and A_{ZF3} exhibit a minimal alteration of the Raman shift with temperatures and become invisible upon approaching 220 K from below, owing to the pronounced CDW fluctuations, hence assigned as the zone-folded modes [5].

In Supplementary Fig. 4b, the Raman spectra from 180 K to 280 K (marked by a dashed-red rectangle in Supplementary Fig. 4a) have been enlarged to show the detailed feature of peak evolution. As temperatures increase from 180 K, the 2.25-THz A_{ZF1} peak can be described well by a single Gaussian function (green-shaded area in Supplementary Fig. 4b). A black arrow marks the peak positions of the 2.25-THz A_{ZF1} peak and a single Gaussian function. However, the 2.25-THz A_{ZF1} peak can no longer be described by a single Gaussian function when the temperature is above 220 K, caused by the splitting of the 2.25-THz A_{ZF1} peak and marked by two arrows. This indicates new modes to develop above 220 K rather than only a single mode of 2.25 THz. Therefore, the 2.25-THz A_{ZF1} peak can only be well identified below 220 K in Raman spectra, consistent with the pump-probe results in Fig. 3a.

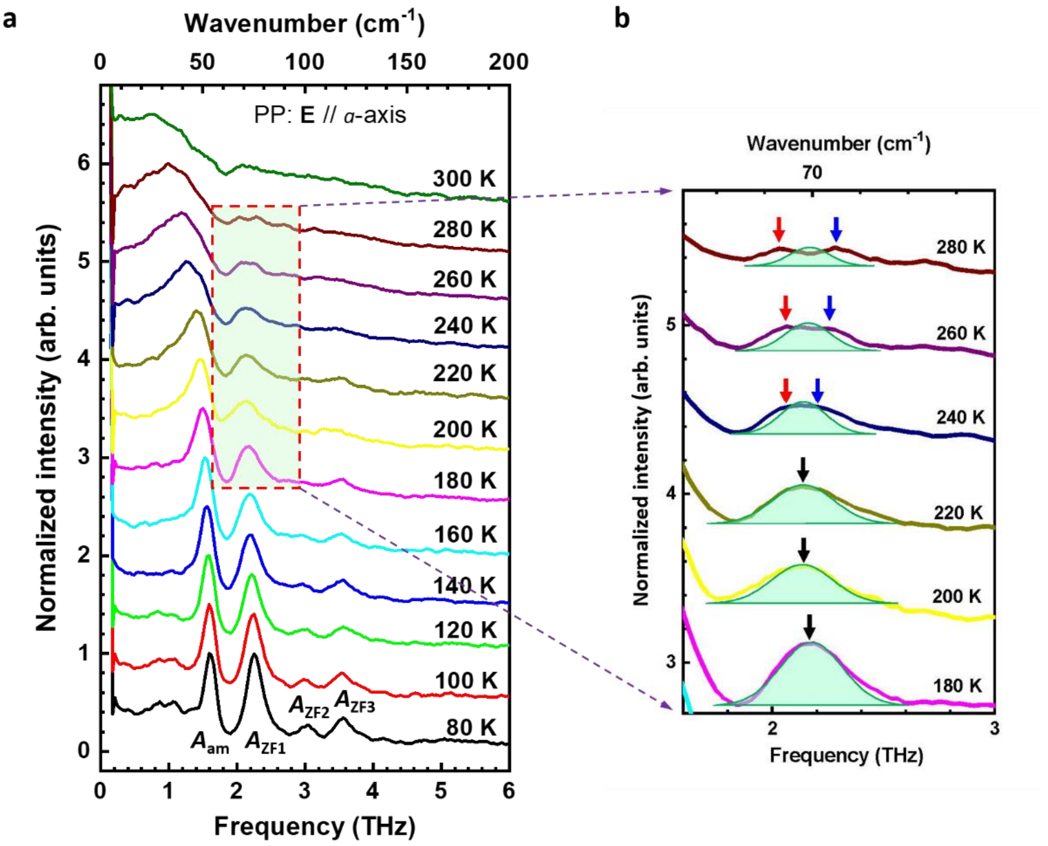

Supplementary Fig. 4. a Raman spectra along the *a*-axis (**E**//*a*-axis) of a CuTe single crystal at various temperatures. PP: The excitation and the detection polarizations were set along the *a*-axis of CuTe crystals. **b** Enlargement of the Raman spectra marked by a dashed-red rectangle in **(a)**. The arrows indicate the positions of peaks, consistent with the peak positions of the Gaussian function (green-shaded area) below 220 K.

Supplementary Note 4: Angle-resolved photoemission spectroscopy (ARPES) of CuTe

ARPES experiments were performed at the beamline BL21B1, National Synchrotron Radiation Research Center (NSRRC) in Taiwan. CuTe single crystals were cleaved in situ and measured at an ultra-high vacuum (UHV) chamber with a base pressure of 5×10^{-11} Torr. In order to study the temperature-dependent evolution of the gap, band dispersions were measured in various temperatures from 100 K to 310 K, slightly below the CDW phase transition temperature of 335 K. Supplementary Fig. 5b displays the band dispersions at 100 K, 180 K and 310 K, respectively, measured parallel to the k_x direction at $k_y = 0.46$ Å^{-1}, where is close to the gap maximum (cut marked with the blue-dashed line in Supplementary Fig. 5a). The linearly dispersing Te P_x bands exhibit a significant CDW gap of 153 meV due to the Te chain modulation [6, 7]. The CDW gap gradually closes as the temperature increases (153 meV/ 100 K to 52 meV/310 K). Supplementary Fig. 5cd exhibits the detailed temperature-dependent gap size extracted from comparing the leading edge of the energy distribution curves (EDCs) at $k_x = 0.41$ Å^{-1} and E_F defined by the reference polycrystalline gold at 80 K. We note that the size of the gap is slightly different from which was reported by previous ARPES experiments [6]. One possible explanation is that the electronic structure of CuTe is predicted to be three-dimensional. In Supplementary Fig. 5, APRES measurements are performed with E_{ph}= 90 eV, i.e., at different k_z from the previous experiments [6]. On top of that, q_{CDW} also exhibits a component in the z direction, which alters the gap size at various k_z.

The green arrow in Supplementary Fig. 6a shows that the ARPES images were performed along the k_z direction from Γ to Z with different photon energies (E_{ph}), which relate to different k_z points. For example, the E_{ph} was tuned from 90 eV to 106 eV (i.e., $k_z = 12.09$ π/c), and the band dispersions of CuTe measured along the k_y direction (momentum cut marked by a green-dashed line at $k_x = 0.41$ Å^{-1} in Supplementary Fig. 6b) at 90 K and 300 K, respectively. By the comparison of corresponding EDC at $k_y = 0.46$ Å^{-1} (a green line in Supplementary Fig. 6e) and the leading edge with the reference spectrum of

polycrystalline gold (a black line in Supplementary Fig. 6e), a CDW gap with 178 meV can be clearly observed at 90 K (also marked by the white arrow in Supplementary Fig. 6c), which is closed to the gap size along the k_x direction (i.e., along the *a*-axis in Fig. 3c). This unambiguously indicates that a CDW phase exists at low temperatures and along the k_z direction (i.e., *c*-axis) to show a CDW phase along *c*-axis. Additionally, as shown in Supplementary Fig. 6f, the CDW gap significantly shrinks at 300 K to indicate the closing of the CDW gap along k_z direction (i.e., *c*-axis) above 220 K. Namely, the CDW phase along the *c*-axis becomes weak at high temperatures even though the 1D CDW phase along the *a*-axis is still pronounced as shown in Fig. 3c.

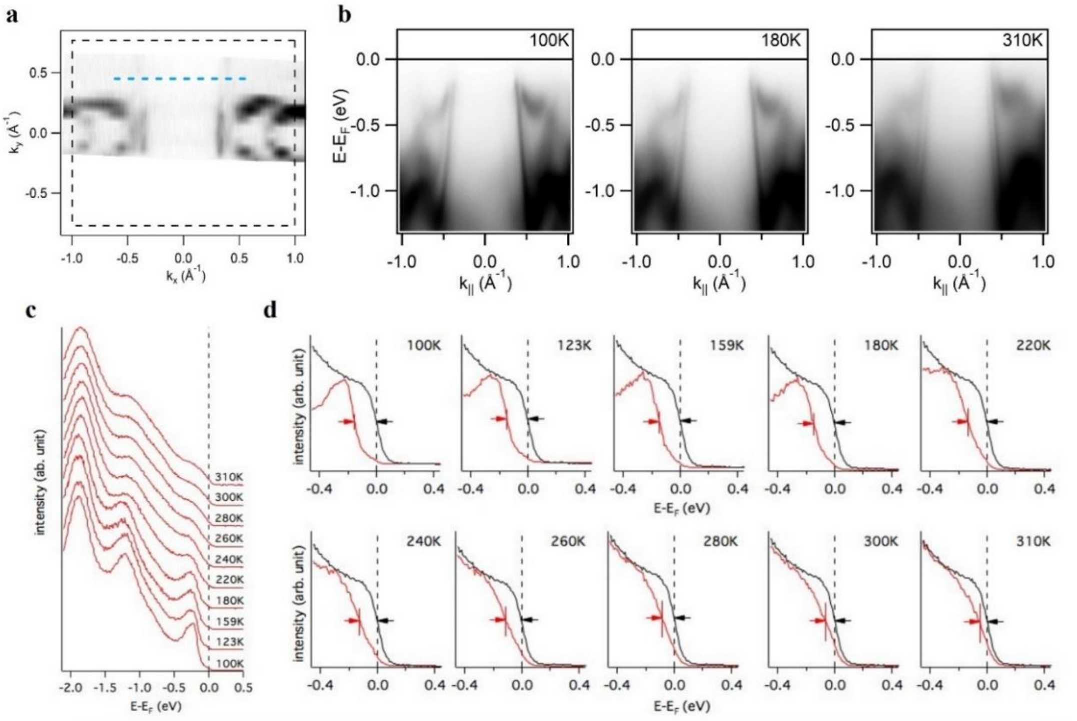

Supplementary Fig. 5. a Fermi surface mapping in k_x-k_y plane measured at 85 K and E_{ph}= 90 eV, p-polarized. **b** Band dispersions of CuTe measured along the k_x direction with E_{ph}= 90 eV at k_y = 0.46 Å^{-1} (momentum cut marked by a blue-dashed line in **(a)** measured at 100 K, 180 K, and 310 K, respectively. **c** Temperature-dependent EDCs at (k_x, k_y) = (0.41, 0.46) Å^{-1} measured with E_{ph}= 90 eV. **d** The comparison of corresponding EDCs at k_x = 0.41 Å^{-1} (red lines) and the leading edge with the reference spectra of polycrystalline gold at 80 K (black). The height of the edges is normalized. The extracted gap size is plotted in Fig 3c.

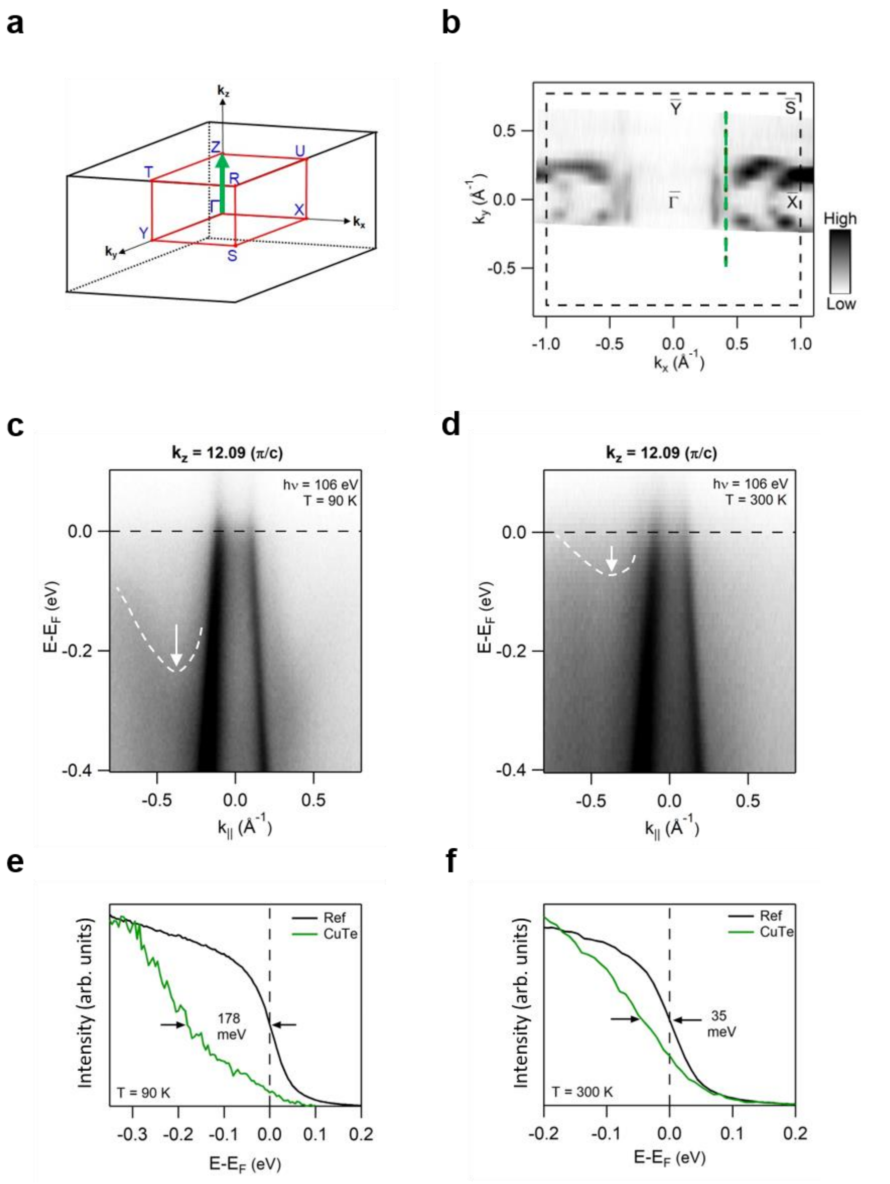

Supplementary Fig. 6. a Illustration of the bulk Brillouin zone of CuTe with high symmetry labels. **b** Fermi surface mapping in the k_x-k_y plane was measured at 85 K and E_{ph}= 90 eV. The dashed rectangle indicates the bulk Brillouin zone with high symmetry labels. **c**, **d** Band dispersions of CuTe measured along the k_y direction (momentum cut marked by a green-dashed line at k_x = 0.41 Å^{-1} in **(b)**) with E_{ph}= 106 eV (which is related to the higher k_z point along the arrow from Γ to Z in **(a)**) at 90 K and 300 K, respectively. **e**, **f** The comparison of corresponding EDCs at k_y = 0.46 Å^{-1} (green lines) and the leading edge with the reference spectra of polycrystalline gold (black lines) at 90 K and 300 K, respectively, where the maximum CDW gap is located. The locations of the maximum CDW gap are indicated by the white arrows in the dispersions.

Supplementary Note 5: Time-dependent Ginzburg Landau (TDGL) equations

To further understand the dynamics of the CDW order in our system, we simultaneously solve the time-dependent Ginzburg-Landau (TDGL) equations for both the electronic density and the atomic configuration [8] derived from the following Landau functional:

$$F[A,u] = -a|A|^2 + \frac{b}{2}|A|^4 - \eta(Au^* + A^*u) + K|u|^4 \quad (1)$$

where A and u are amplitudes for the electronic density modulation and the ion displacement, respectively. Expressly, one first assumes that the time dependence of these amplitude modes exhibits a damped oscillatory behavior with frequency ω_{AM} and damping parameter γ_{AM}, and from TDGL equations, one obtains a characteristic cubic polynomial equation for $\Lambda \equiv \omega_{AM} + i\gamma_{AM}$:

$$P(\Lambda) = \tau_0\Lambda^3 + \left(2\Theta + \zeta + \gamma_y\omega_0\tau_0\right)\Lambda^2 + \omega_0\left(2\Theta\gamma_y + \zeta\gamma_y + \omega_0\tau_0\right)\Lambda + 2\Theta\omega_0^2 = 0 \quad (2)$$

As shown in Fig. 3a, we use the solutions of Supplementary Eq. (2) to fit our measured Λ (i.e., the frequency of the oscillation component of $\Delta R/R$) to obtain the electronic density relaxation time τ_0, CDW phonon frequency ω_0, CDW phonon damping coefficient γ_y, and the CDW phase transition temperature T_c. Here $\zeta \equiv 1 - T_0/T_c$ is related to the unrenormalized critical temperature T_0 [8] and $\Theta \equiv 1 - T/T_c$.

We then use the above-fitted parameters to solve TDGL equations [8] with a time-dependent temperature T to compare our time-dependent electronic CDW order with theory. Here, the time dependence of the temperature is computed using the following parameters: the pump photon energy of 3.1 eV, the absorbed photon volume density of 0.168×10^{20}, molar volume 26.92 cm^3 [9], heat capacity of the lattice $C_{tot} = 49.49$ mJ mol^{-1}K^{-1} [10], and the fraction of hot phonons $\kappa = 0.5$. The low- and high-temperature electronic heat

capacities are given by $c_0 = 1.43\ \mathrm{mJ\ mol^{-1}K^{-2}}$ and $c = 4\ \mathrm{mJ\ mol^{-1}K^{-2}}$ [8], respectively. The exchange rates between electrons and hot phonons and that between electrons and the other phonons are given by $5.5\ \mathrm{J\ ps^{-1}K^{-1}\ mol^{-1}}$ and $7.25\ \mathrm{J\ ps^{-1}K^{-1}\ mol^{-1}}$, respectively.

In Fig. 2c and 2d, we plot the Landau free energy for the modulation of the electronic density. For the illustrative purpose, the lattice order is taken to be proportional to the electronic order according to $u = \frac{\eta}{K}A$. As shown in Fig 2c, the double well potential structure is developed very quickly at a low-temperature $T = 37\ \mathrm{K}$ and is more pronounced than at a high-temperature $T = 280\ \mathrm{K}$ as in Fig 2d. As a result, the CDW order exhibits many oscillations in time at the low temperature (Fig. 2c) while it is damped to the equilibrium value quickly at the high temperature (Fig. 2d).

Supplementary Note 6: First-principles electronic structure calculations

Based on the density functional theory (DFT), the first-principles calculations for the electronic structures of quasi-one dimensional CuTe were performed using the Quantum Espresso code [11] with the ultrasoft pseudopotentials. The generalized gradient approximation in the Perdew-Burke-Ernzerhof (PBE) functional [12] was employed to approximate the exchange-correlation interaction. An energy cutoff of 45 Ry was adopted to expand the plane-wave basis of the wavefunction. The experimental crystal structure of the primitive cell (orthorhombic, Pmmn, a = 3.1537 Å, b = 4.0933 Å, c = 6.9621 Å) was used. Atomic configurations of possible CDW modulations, CDW along the a-axis (CDW_a) and CDW along the c-axis (CDW_c), guided by the eigenvectors of the soft modes with calculated imaginary phonon frequencies along the Γ-X and Z-U of the normal-phase Brillouin Zone (BZ) were respectively optimized in the 5×1×1 and 5×1×2 supercells. The convergence criteria of all atomic forces are better than 10^{-5} Ry/Bohr. The k-point grid of the normal phase with a primitive unit cell (20×16×8), CDW_a (4×16×8), and CDW_c (4×16×4) were set to perform corresponding BZ integrations. In order to explore the lattice dynamics of CuTe, phonon dispersions of the normal, CDW_a, and CDW_c were also calculated on the q grids of 10×4×2, 2×4×2 and 2×4×1, respectively, within the framework of density functional perturbation theory (DFPT) [11]. Moreover, the effects of CDW modulation on the electronic band structures and phonon dispersions were investigated by the unfolding method implemented in a modified unfold-x code [13]. The wave character of the charge density in a CDW phase was clarified via the charge density difference (ρ_{diff}) between the CDW and normal (non-CDW) phases, $\rho_{\mathrm{diff}} = \rho_{\mathrm{CDW}} - \rho_{\mathrm{normal}}$.

According to our calculated total energies, CDW_a and CDW_c with ΔE= -0.7 and -1 meV/atom, respectively, compared to the normal phase, exhibit the stabilities of two CDW structures through the competition between corresponding electronic and elastic energies. Specifically, as shown in Fig. 3d and 3e in the main text, the individual lattice distortion of

adjacent CuTe layers in CDW_a could be coupled to form a 3D-like structural modulation along the *c*-axis of CDW_c. The non-negligible energy difference (0.3 meV/atom) between two CDWs indicates that interlayer interactions will play a key role in the ground-state regime of CuTe crystals.

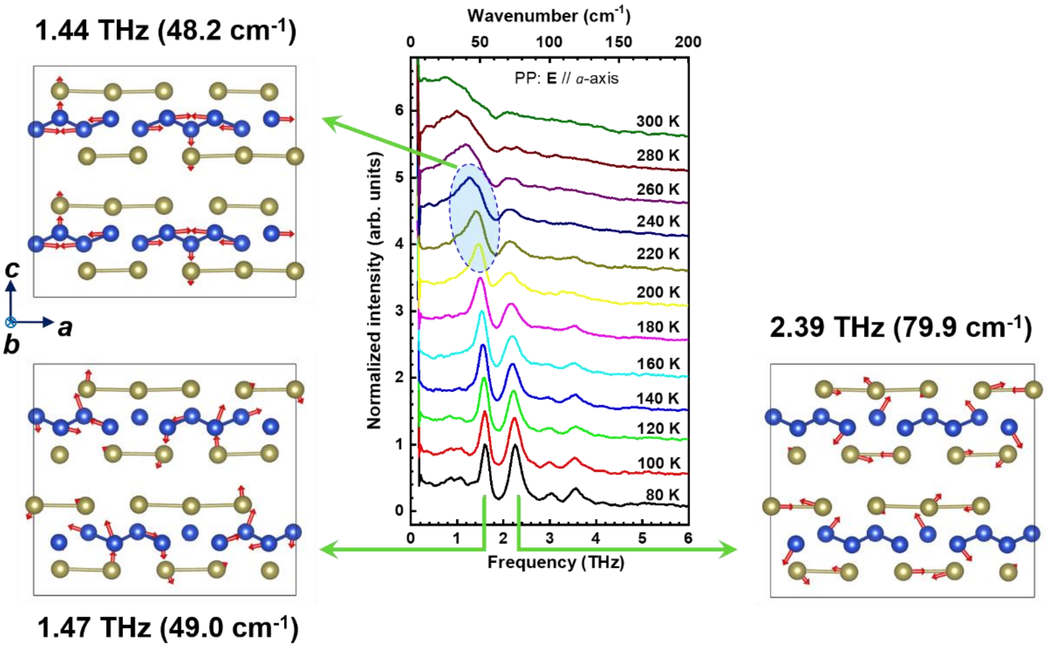

Supplementary Fig. 7. The measured Raman data (Supplementary Fig. 4a) and corresponding calculated eigenvectors of amplitudon-like modes in both CDW_a and CDW_c. Copper and tellurium atoms are denoted as blue and golden spheres, where red arrows depict atomic displacements.

In fact, the eigenvector of lattice vibrations can be treated as a fingerprint of the interlayer coupling. As shown in Supplementary Fig. 7, the eigenvector of the amplitudon-like mode (1.44 THz/48.2 cm^{-1}) dominated by in-plane displacements of copper atoms along the *a*-axis indicates a quasi-1D feature of CDW_a. On the other hand, an increase in interlayer interaction in CDW_c was identified by significant out-plane components of corresponding atomic displacements of copper and tellurium atoms in both modes (1.47 THz/49.0 cm^{-1} and 2.39 THz/79.9 cm^{-1}). Furthermore, the electronic charge density contour of the computationally optimized atomic configuration (as shown in Fig. 3h in the main text)

indeed shows a CDW modulation of the building block along the *a*-axis of CuTe crystals in which the new periodicity of Te charge density is consistent with the line profile of STM image (Fig. 3g in the main text).

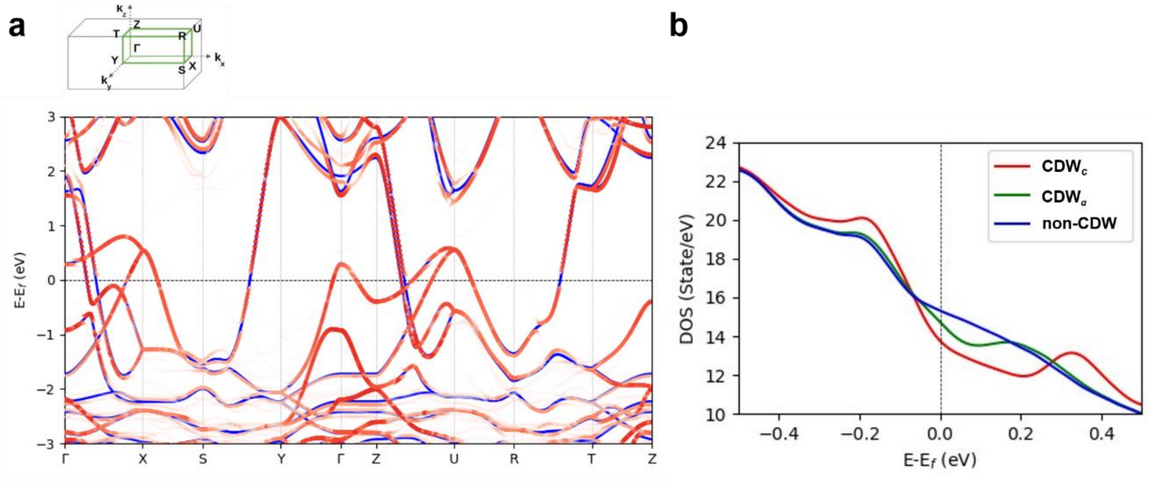

Supplementary Fig. 8. a Calculated band structure of normal phase (blue curves) and unfolded band structure with unfold weights of CDW_c (red curves with grey scales) of CuTe crystals. Energies are aligned with the Fermi level (E_F), denoted as the black dashed line. The inset illustrates the Brillouin Zone (BZ). **b** Density of state (DOS) of CDW_c, CDW_a, and non-CDW phases around E_F.

Interestingly, using PBE functional at experimental lattice constants (a = 3.1537 Å, b = 4.0933 Å, c = 6.9621 Å), visible partial gap openings can be found along the k_x direction of the BZ in the unfolding band structure (red curves) of CDW_c and the band structure (blue curves) of normal CuTe, as shown in Supplementary Fig. 8. The band gap values from the Fermi level (E_F) opening at k_x= 0.2a* along the Γ-X, Y-S, Z-U, and T-R of the BZ are 0.400, 0.140, 0.400, and 0.137 eV, respectively. Such considerable energy gains could overwhelm the elastic energy costs and trigger the CDW lattice distortions. The slightly overestimated gap size compared with previous results [6] could be attributed to the thermal effects not considered in our calculations.

The calculated phonon dispersion curves of CuTe in a normal phase/CDW_c are presented in blue/red curves, respectively, in Supplementary Fig. 9. Significant phonon softening with imaginary frequencies around $q_1 = (0.4, 0.0, 0.0)$ and $q_2 = (0.4, 0.0, 0.5)$ along the Γ-X and Z-U, respectively, reveal a structural instability of CuTe normal phase. However, the renormalized phonon spectrum with all real frequencies unfolded from a 5×1×2 supercell calculation demonstrates that such structural modulation will stabilize the CDW_c of CuTe.

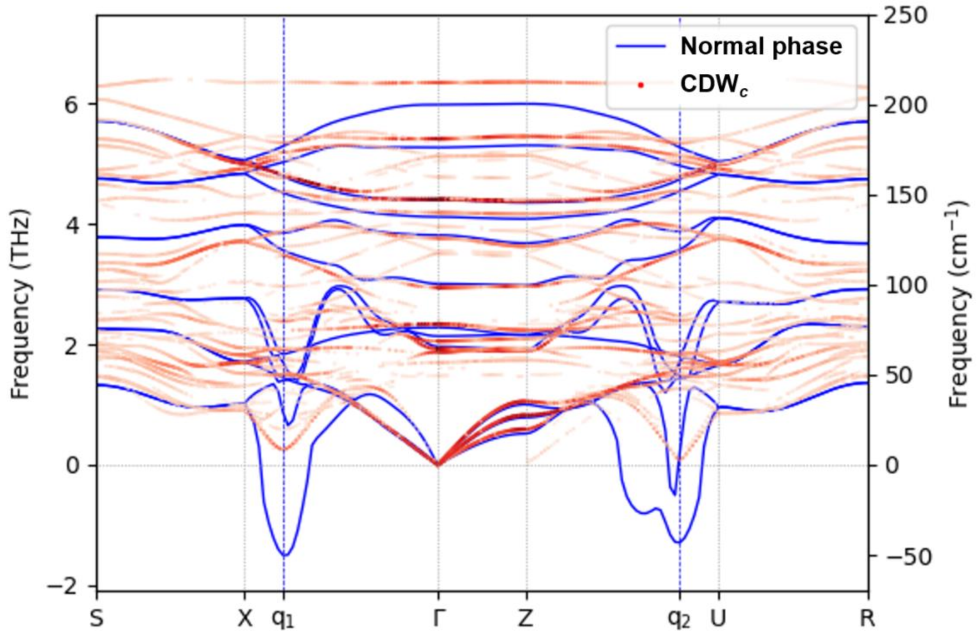

Supplementary Fig. 9. DFPT calculated phonon spectrum of CuTe in the normal phase (blue curves) and CDW_c (red curves). The corresponding unfold weight is shown on a grey scale. Phonon soft modes with prominent imaginary frequencies are indicated as q_1 (0.4, 0.0, 0.0) and q_2 (0.4, 0.0, 0.5) along the Γ-X and Z-U paths, respectively.

To examine the *c*-axis character of electronic charge modulation in the CDW phase, we further calculated the charge density difference of two adjacent building blocks of CuTe layers, as shown in Fig. 4d in the main text. A periodic charge fluctuation mainly formed around copper atoms was clearly observed in the upper CuTe layer (red dashed rectangle). Meanwhile, the anti-phase of the charge distortion was established in the next CuTe layer, reflecting the 3D nature of CDW_c modulation.

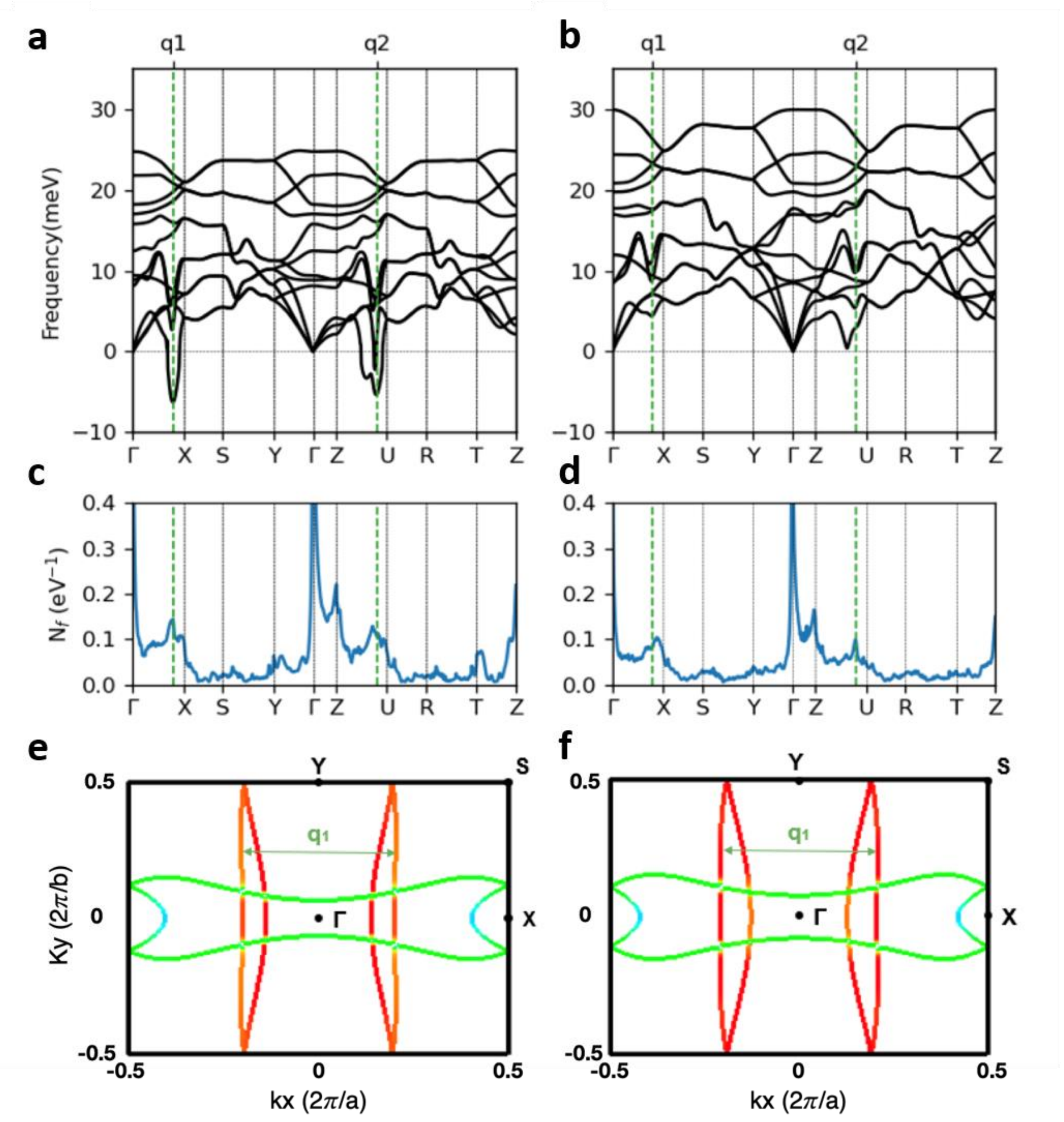

Supplementary Fig. 10. The DFPT calculated phonon spectra of the non-CDW phase of CuTe at **a** ambient pressure and **b** 9 GPa. The nesting factors/Fermi surface contours at $k_z = 0$ are shown in **c**/**e** and **d**/**f** for ambient and high-pressure (9 GPa) conditions, respectively. Dark green dashed lines indicate the CDW ordering q-vectors (q_1 and q_2).

Moreover, we have calculated Fermi surfaces, a nesting factor which is the low-frequency limit of the bare electronic susceptibility, and phonon spectra of the non-CDW phase under ambient and high (9 GPa) pressure. As shown in Supplementary Fig. 10ab, all the imaginary frequencies of the phonon spectrum at ambient conditions can be stabilized.

Therefore, the corresponding CDW instability can be destroyed under external hydrostatic compression. However, both the peak features at CDW ordering q-vectors [q_1 = (0.4, 0, 0) and q_2 = (0.4, 0, 0.5) along the Γ-X and Z-U paths, respectively in Supplementary Fig. 10cd] of the nesting factors (N_f) and corresponding contours of FS topologies, as illustrated in Supplementary Fig. 10ef, demonstrated the nature of nesting is preserved in both non-CDW and CDW conditions. Therefore, the purely electronic FSN is not the main driving mechanism responsible for developing CDW modulations in CuTe.

For the possible thermal effects on the stability of two soft modes, it is well known that fully finite-temperature first-principles calculations, especially for the CDW modulations, including large-scale supercells, are extremely computational demanding, which is out of the scope of the present work. However, it is still possible to explore qualitatively thermal effects by performing calculations in different smearing widths for various artificial electronic temperatures and corresponding lattice constants. As shown in Supplementary Fig. 11a, it is clear that the soft mode at q_1 (0.4, 0, 0) corresponding to the CDW_a is more sensitive to increasing smearing width (artificial temperature) and could be stabilized, while the mode at q_2 (0.4, 0, 0.5) responsible for the CDW_c is still softening. Also, the calculated phonon spectra of the non-CDW phase of experimental lattice constants at different temperatures, as illustrated in Supplementary Fig. 11b, show the similar thermal behaviors of the two modes at q_1 and q_2, respectively. Furthermore, the unfolded phonon spectrum of the CDW_a state in Supplementary Fig. 11c supports that such 5×1×1 supercell modulation could only stabilize the soft mode at q_1 rather than the one at q_2. Indeed, as discussed in Supplementary Fig. 9, only CDW_c modulation derived from the eigenvector of the imaginary frequency at q_2 of the non-CDW phase could stabilize all the phonon instabilities.

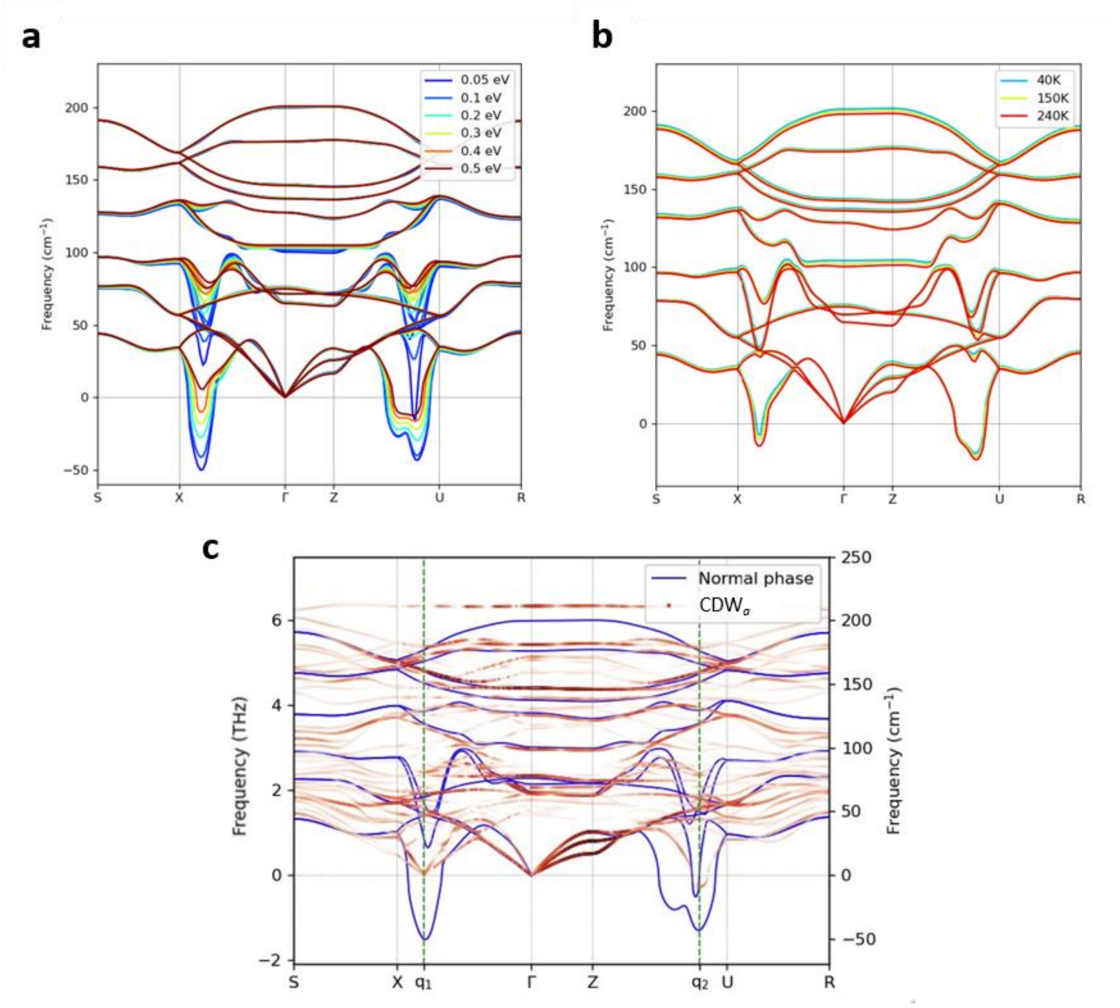

Supplementary Fig. 11. The DFPT calculated phonon spectra at room-temperature lattice constants and different smearing widths **a** and lattice coordinates at different temperatures **b** of the non-CDW phase of CuTe. **c** The DFPT calculated phonon spectra of CuTe in normal phase (blue curves) and CDW$_a$ (red curves). The corresponding unfold weight is shown on a grey scale. Phonon soft modes with prominent imaginary frequencies are indicated as q_1 (0.4, 0.0, 0.0) and q_2 (0.4, 0.0, 0.5), respectively.

Supplementary Note 7: Configuration of θ-dependent $\Delta R/R$ spectra in Fig. 4a

Supplementary Fig. 12a represents the configuration of a CDW probed by a probe beam with various incident angles θ. First, in the normal incident case with $\theta = 0°$, a propagation direction of probe pulses **k**//c-axis and electric field **E**//a-axis ($\perp$ c-axis and $\mathbf{P}_c$). Here, the $\mathbf{P}_c$ is an electric dipole, which is caused by the displacements of Cu and Te atoms along the c-axis, as shown in Supplementary Fig. 7. Thus, there is no electric field **E** to interact with the $\mathbf{P}_c$ according to the relation of $\Delta R/R_c \propto \mathbf{E}\cdot\mathbf{P}_c = |\mathbf{E}||\mathbf{P}_c|\cos(90\text{-}\theta) = 0$ with $\theta = 0°$. For the case of oblique incidence, the **E** field of probe pulses can be decomposed into two perpendicular components: $\mathbf{E}_1$ and $\mathbf{E}_2$. $\mathbf{E}_2$ is parallel to the a-axis and $\mathbf{P}_a$ (caused by the displacements of Cu and Te atoms along the a-axis as shown in Supplementary Fig. 7), and $\mathbf{E}_1$ is parallel to the c-axis and $\mathbf{P}_c$, which discloses the CDW along the c-axis with a wavevector of q_c ($\because q_c$ // $\mathbf{P}_c$). Under this configuration, the $\Delta R/R_c \propto \mathbf{E}\cdot\mathbf{P}_c = |\mathbf{E}||\mathbf{P}_c|\cos(90\text{-}\theta) \neq 0$ with $\theta \neq 0°$ and the $\Delta R/R_c$ will become larger when increasing the incident angle θ as demonstrated by Fig. 4b in the main text. Meanwhile, the effective propagation vector of $\mathbf{E}_1$ is $\mathbf{k}_1$, which increases with increasing the incident angle θ. The data at small incident angles of $\theta < 30°$ in Supplementary Fig. 12c are almost the same frequency. When the θ increases to 45°, however, the frequency drops significantly from ~2.3 THz to ~1.97 THz.

In order to figure out this issue, we further enlarged the DFPT calculated phonon spectra of CuTe in the CDW$_c$ phase around Γ point in Supplementary Fig. 9, as shown in the inset of Supplementary Fig. 12c. Actually, there are many phonon bands near Γ point, including A_g, B_{1g}, B_{2g}, B_{3g}, B_{1u}, B_{2u}, and B_{3u} modes (red/green curves in the inset of Supplementary Fig. 12c). But only the A_g mode with c-axis-component eigenvectors (i.e., $\mathbf{P}_c$, see Supplementary Figs. 12efg) can be detected by our linear-polarized probe beam according to $\Delta R/R_c \propto \mathbf{E}\cdot\mathbf{P}_c$. Besides, the $A_{g,1}$ mode has the largest $\mathbf{P}_c$. Therefore, the $A_{g,1}$

mode with a frequency of ~2.39 THz would dominate the $\Delta R/R_c$ spectra at small incident angles with a small $\mathbf{k}_l$ vector of the probe beam, e.g., the dashed line (photon dispersion) in the inset of Supplementary Fig. 12c. For the larger incident angles, the photon dispersion line (e.g., dot-dashed line in the inset of Supplementary Fig. 12c) may cross with both $A_{g,2}$ and $A_{g,3}$ modes, but not $A_{g,1}$ mode. However, the c-axis-component eigenvectors of $A_{g,2}$ mode is rather smaller than that of $A_{g,3}$ mode as shown in Supplementary Figs. 12f and 12g. Consequently, the $A_{g,3}$ mode with a frequency of 1.93 THz could be detected, which agrees with our data point (~1.97 THz) at 45°.

Once the **E** field of the probe pulses was set to parallel with the b-axis as the configuration in Supplementary Fig. 12b, there is only ~1.61-THz peak in all spectra with different incident angles (see Supplementary Fig. 12d). Therefore, the A_{HF} peaks with ~2.31 THz observed in the incident angle-dependent spectra are polarization-sensitive and from the same mode. Additionally, the $\Delta R/R_a \propto \mathbf{E}\cdot\mathbf{P}_a$ and $\Delta R/R_b \propto \mathbf{E}\cdot\mathbf{P}_b$ can also be realized via the polarization rotation of probe pulses on ab-plane, respectively. This technique has been applied to study the dichotomy of photoinduced quasiparticle dynamics in high T_c superconductors $YBa_2Cu_3O_7$ [14-16]. The d-wave symmetry of the superconducting gap and pseudogap in high T_c superconductors was successfully revealed by the distribution of the temperature evolution of $\Delta R/R$ on the CuO_2 planes of $YBa_2Cu_3O_7$. This indicates that the orientation-/time-resolved ultrafast spectroscopy developed in this study can reveal the ordering or order parameters in various orientations (dimensions) in materials.

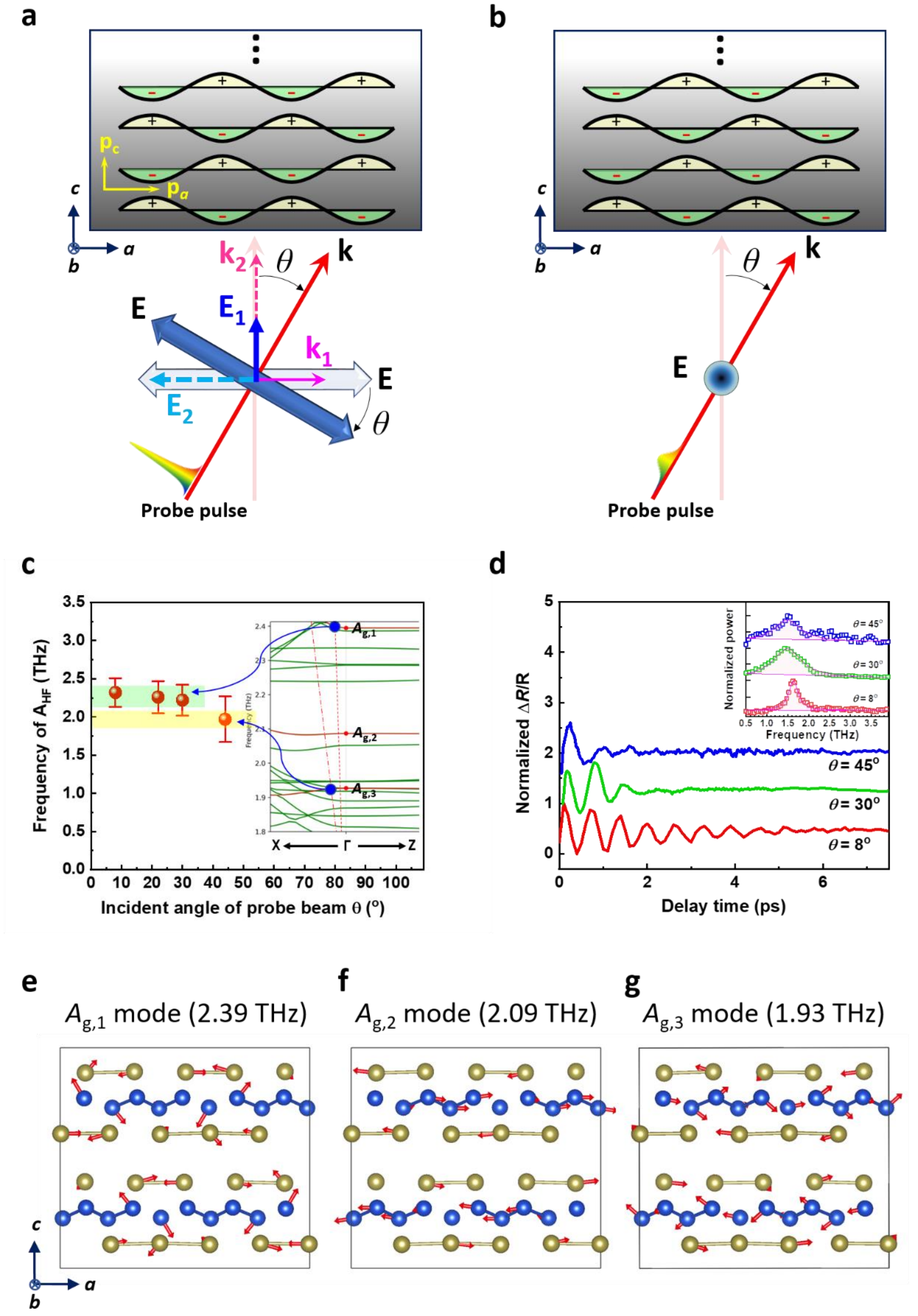

Supplementary Fig. 12. a Schematics of the configuration of a CDW phase probed by a probe pulse with various incident angles θ and electric field **E**⊥b-axis. $\mathbf{E}_1$ (//c-axis and //$\mathbf{P}_c$) and $\mathbf{E}_2$ (//a-axis and //$\mathbf{P}_a$) are the components of the electric field **E** of a probe pulse. **k** is the

propagation direction of the probe pulses. $\mathbf{k}_1$ (//a-axis and ⊥$\mathbf{P}_c$) and $\mathbf{k}_2$ (//c-axis and ⊥$\mathbf{P}_a$) are the components of the propagation direction **k** of probe pulses. **b** Schematics of a CDW phase probed by a probe pulse with various incident angles θ and electric field **E**//b-axis. **k** is the propagation direction of the probe pulses. **c** Frequency of A_{HF} peak in Fig. 4b as a function of incident angles θ of the probe pulses. Inset: Enlargement of the DFPT calculated phonon spectrum of CuTe in CDW$_c$ phase around Γ point in Supplementary Fig. 9. Red curves indicate the dispersion of $A_{\mathrm{g},1}$, $A_{\mathrm{g},2}$, and $A_{\mathrm{g},3}$ modes. Green curves show the $B_{1\mathrm{g}}$, $B_{2\mathrm{g}}$, $B_{3\mathrm{g}}$, $B_{1\mathrm{u}}$, $B_{2\mathrm{u}}$, and $B_{3\mathrm{u}}$ modes, respectively. Red dashed and dot-dashed lines are the photon dispersion. The error bars are obtained from the standard deviation of the least square fitting. **d** Normalized oscillation components in $\Delta R/R$ spectra of a CuTe single crystal at different incident angles, θ (ϕ_1 and $\phi_2 = 90°$, i.e., the configuration in **(b)**) at $T = 60$ K. Inset: Fourier transform spectra of the oscillation spectra in **(d)**. **e**, **f, g** Calculated eigenvectors of $A_{\mathrm{g},1}$, $A_{\mathrm{g},2}$, and $A_{\mathrm{g},3}$ modes in the inset of **(c)**. Copper and tellurium atoms are denoted as blue and golden spheres, where red arrows depict atomic displacements.

Supplementary Note 8: Characterizations of CuTe crystal structure

The high-quality CuTe single crystals used in this study are characterized by x-ray diffraction (XRD), as shown in Supplementary Fig. 13a, which was measured at TPS 09A in NSRRC with an x-ray energy of 12 keV (λ = 1.0332 Å). The (00ℓ) plane is a natural cleavage facet of as-grown single crystals. The extracted lattice parameters are a = 3.1537 Å, b = 4.0933 Å, and c = 6.9621 Å, in agreement with previous reports [10, 17]. The energy-dispersive x-ray (EDX) spectroscopy data in Supplementary Fig. 13b gives the ratio of Cu:Te as 1.02 (1):1.

According to the temperature versus composition phase diagram of the Cu-Te system, there are various polymorphic phases and crystal structures [18]. For example, the superionic Cu_2Te has a structure where the tellurium atom forms a rigid sublattice, and the liquid-like Cu ions are distributed randomly in the rigid sublattice and thus classified as phonon-liquid-electron-crystals. Additionally, the short phonon lifetime and small lattice thermal conductivity in Cu_2Te have been revealed by Raman spectroscopy with rich Raman modes below 200 cm^{-1} [19]. Even though there are no significant Raman modes for CuTe at room temperature, the feature of plasmon-phonon coupling can be observed in a low wavenumber regime (<60 cm^{-1}) as shown in Supplementary Fig. 4.

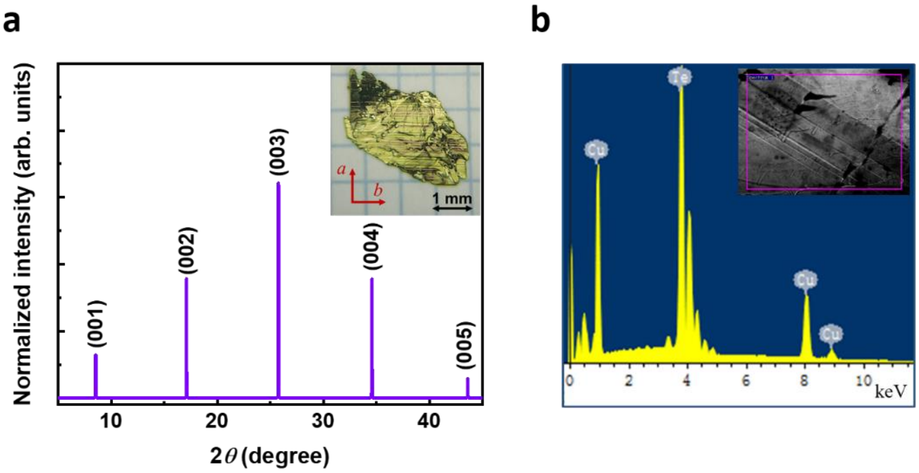

Supplementary Fig. 13. a X-ray diffraction pattern of one CuTe single crystal at 300 K. Inset: a photo of one as-grown CuTe single crystal. **b** Energy-dispersive x-ray spectroscopy of one CuTe single crystal.

Non-resonant x-ray diffraction was employed to measure the CDW peak with the modulation vector q_{CDW} = (0.4, 0, 0.5). The acquisition of sufficient data necessitates the high-brightness synchrotron x-ray source. Therefore, all the non-resonant XRD measurements were executed at TPS 09A in NSRRC with an x-ray energy of 12 keV (wavelength = 1.0332 Å). The CuTe single crystal was mounted in a closed-cycle cryostat on a six-circle Huber diffractometer, enabling temperature control from 100 K to 300 K. Supplementary Fig. 14a shows that the L-scan across CDW modulation vector q_{CDW} = (0.4, 0, 0.5) were measured along the (0, 0, L) direction of one CuTe single crystal at different temperatures. The (0, 0, 0.5L) peak significantly disappears while $T > 200$ K, which clearly indicates the phase transition along the c-axis accompanying the c-axis doubling (i.e., 1c unit → 2c unit) at low temperatures. This change of spatial periodicity further creates zone-folded (ZF) modes near the zone center (see the Raman spectra in Supplementary Fig. 4). Moreover, the (0.4, 0, 2) peak can still be observed at 300 K, as shown in Supplementary Fig. 14b. This demonstrates that the $q_{\mathrm{CDW}} = 0.4a^*$ can subsist at $T > 200$ K, even if there is no $q_{\mathrm{CDW}} = 0.5c^*$.

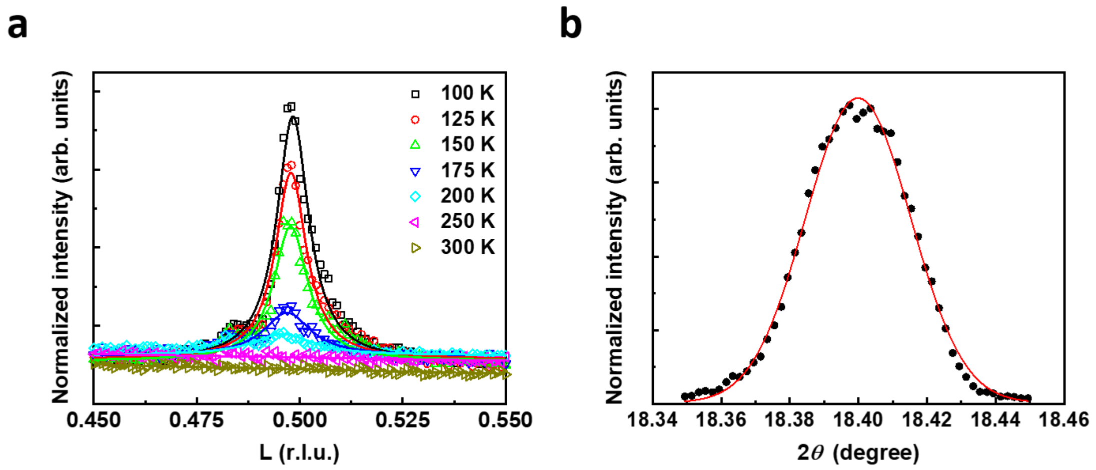

Supplementary Fig. 14. **a** L-scan across CDW modulation vector q_{CDW} = (0.4, 0, 0.5) was measured along the (0, 0, L) direction of one CuTe single crystal at different temperatures, indicating the phase transition along the c-axis. **b** The x-ray diffraction (θ-2θ scan) peak of (0.4, 0, 2) at 300 K.

Supplementary References

1. Dolgirev, P. E. et al. Amplitude dynamics of the charge density wave in $LaTe_3$: Theoretical description of pump-probe experiments. *Phys. Rev. B* **101**, 054203 (2020).
2. Schaefer, H., Kabanov, V. V. & Demsar, J. Collective modes in quasi-one-dimensional charge-density-wave systems probed by femtosecond time-resolved optical studies. *Phys. Rev. B* **89**, 045106 (2014).
3. Yusupov, R. et al. Coherent dynamics of macroscopic electronic order through a symmetry breaking transition. *Nat. Phys.* **6**, 681-684 (2010).
4. Tanaka, T., Harata, A. & Sawada, T. Subpicosecond surface-restricted carrier and thermal dynamics by transient reflectivity measurements. *J. Appl. Phys.* **82**, 4033-4038 (1997).
5. Wang, S. Y. et al. Observation of room-temperature amplitude mode in quasi-one-dimensional charge-density-wave material CuTe. *Appl. Phys. Lett.* **120**, 151902 (2022).
6. Zhang, K. et al. Evidence for a quasi-one-dimensional charge density wave in CuTe by angle-resolved photoemission spectroscopy. *Phys. Rev. Lett.* **121**, 206402 (2018).
7. Kim, S., Kim, B. & Kim, K. Role of Coulomb correlations in the charge density wave of CuTe. *Phys. Rev. B* **100**, 054112 (2019).
8. Dolgirev, P. E. et al. Amplitude dynamics of the charge density wave in $LaTe_3$: Theoretical description of pump-probe experiments. *Phys. Rev. B* **101**, 054203 (2020).
9. Molar volume: $\nu = M/\rho$. (M & ρ from https://www.americanelements.com/copper-ii-telluride-12019-23-7)
10. Kuo, C. N., Huang, R. Y., Kuo, Y. K. & Lue, C. S. Transport and thermal behavior of the charge density wave phase transition in CuTe. *Phys. Rev. B* **102**, 155137 (2020).
11. Giannozzi, P. et al. Quantum ESPRESSO toward the exascale. *J. Chem. Phys.* **152**, 154105 (2020).

12. Perdew, J. P., Burke, K. & Ernzerhof, M. Generalized gradient approximation made simple. *Phys. Rev. Lett.* **77**, 3865 (1996).

13. Popescu, V. & Zunger, A. Extracting E versus k effective band structure from supercell calculations on alloys and impurities. *Phys. Rev. B* **85**, 085201 (2012).

14. Luo, C. W. et al. Dichotomy of photoinduced quasiparticle on CuO_2 planes of $YBa_2Cu_3O_{7-\delta}$ directly revealed by femtosecond polarization spectroscopy. *J. Appl. Phys.* **102**, 033909 (2007).

15. Luo, C. W. et al. Spatial dichotomy of quasiparticle dynamics in underdoped thin-film $YBa_2Cu_3O_{7-\delta}$ superconductors. *Phys. Rev. B* **74**, 184525 (2006).

16. Luo, C. W. et al. Spatial symmetry of superconducting gap in $YBa_2Cu_3O_{7-\delta}$ obtained from femtosecond spectroscopy. *Phys. Rev. B* **68**, 220508R (2003).

17. Stolze, K. et al. CuTe: Remarkable bonding features as a consequence of a charge density wave. *Angew. Chem. Int. Ed. Engl* **52**, 862 (2013).

18. Pashinkin, A. S. et al. Phase equilibria in the Cu–Te system. *Inorg. Mater.* **39**, 539 (2003).

19. Pandey, J. et al. Raman spectroscopy study of phonon liquid electron crystal in copper deficient superionic thermoelectric $Cu_{2-x}Te$. *ACS Appl. Energy Mater.* **3**, 2175 (2020).
